# A genomic region associated with iteroparous spawning phenology is linked with age‐at‐maturity in female steelhead trout

**DOI:** 10.1111/eva.13622

**Published:** 2023-12-11

**Authors:** Stuart Willis, Jeff Stephenson, Andrew Pierce, Lea Medeiros, Laura Jenkins, Douglas R. Hatch, Shawn Narum

**Affiliations:** ^1^ Hagerman Genetics Lab, Columbia River Inter‐Tribal Fish Commission Hagerman Idaho USA; ^2^ Department Biological Sciences University of Idaho Moscow Idaho USA; ^3^ Department Fishery Science, Columbia River Inter‐Tribal Fish Commission Portland Oregon USA

**Keywords:** bioinfomatics/phyloinfomatics, fisheries management, genomics/proteomics

## Abstract

Age‐at‐maturity and iteroparity are two life history variations of steelhead trout (*Oncorhynchus mykiss*) that are believed to increase population resilience and stability. While repeat‐spawning individuals are thought to have historically made up a substantial portion of the reproductive population in the Columbia River and the majority of females still attempt outmigration as kelts, return rates of repeat‐spawner are low throughout the basin and below 1% for the furthest migrating stocks. Notably, outmigrating adults exhibit variation in rematuration phenology, displaying either “consecutive” (reproduce immediately the following season) or “skip” (delay spawning for future seasons) spawning patterns. Here, we use low coverage whole genome sequencing of consecutive versus skip spawning female Columbia River steelhead from two populations to test for genomic differences between these two iteroparous phenotypes. We identified genomic regions on several chromosomes which were associated with the phenology of iteroparity, including a region on chromosome 25 containing two genes, estradiol receptor beta (*ER*β) and glycoprotein hormone beta‐5 (*GPHB5*), which, in mammals, are estrogen‐sensitive and expressed in reproductive tissues. Allele frequencies in this *ER*β/*GPHB5* region differed among female steelhead of different age at maturity, but not males. These genes also shared an island of linkage disequilibrium with the *SIX6* gene, 600Kbp away on the same chromosome, a region of known association with age‐at‐maturity. These observations contribute to growing evidence that age‐at‐maturity and the phenology of iteroparity are determined by overlapping physiological processes and genetic pathways.

## 1 INTRODUCTION

Species exhibit suites of life history traits that vary across space and time. In cases where the variance in these traits is negatively correlated or uncorrelated, these varying suites may have the effect of buffering the population from predictable or stochastic perturbations in a phenomenon called “portfolio effects” (Hoelzel et al., 2019; Moore et al., 2014; Schindler et al., 2010, 2015). Even at the family level, producing offspring with different combinations of life history traits represents a form of bet‐hedging against an unpredictable environment, and may thus be favored as an evolutionary strategy (Schindler et al., 2015). Few salmonid species exhibit as much life history variation as *Oncorhynchus mykiss*, commonly called rainbow trout, redband trout, or steelhead. Variation in life history includes adult migration phenology (run timing), age‐at‐maturity (age at first return migration), precocial sexual maturation (sneaker males), timing of smoltification (age at first seaward migration), propensity for residency or anadromy, and semelparity versus iteroparity (repeat spawning) (Busby et al., 2000; Carlson & Seamons, 2008; Quinn et al., 2011). Among these variations, iteroparity and variation in age‐at‐maturity and have been identified as providing large benefits for population stability: variation in age‐at‐maturity reduces the correlation in population dynamics among years, while iteroparous individuals spawn in multiple years and further erode the association between cohort and spawn year (Berg et al., 1998; Christie et al., 2018; Hatch et al., 2013; Keefer et al., 2008, 2018; Moore et al., 2014). *Oncorhynchus mykiss* which migrate to the ocean (smoltify) will usually spend one to two years in the river and then one or more years in the ocean before returning to spawn (as steelhead), and while variation for age‐at‐maturity within populations is high, some populations, like those in the Clearwater River in the Snake Basin, show strong propensity for longer ocean duration and larger size at return (Bowersox et al., 2019; Copeland et al., 2017; Keefer et al., 2008). Also, unlike most of their congeners, post‐spawn mortality (semelparity) is not biologically programmed in steelhead, and many may try to migrate back to the ocean and return to spawn again in the following (consecutive spawning) or future (skip spawning) seasons. While the historical proportions of fish which attempt to return to the ocean after spawning was estimated to be substantial (17%–46%; Hatch et al., 2013 and references therein), contemporary outmigration survival rates have been estimated to be <10% for Snake River inland stocks because of the numerous impediments, with survival favored for females, younger and smaller fish, and those of non‐hatchery origin (Keefer et al., 2008, 2018). For populations under threat of extirpation in the inland region of the Columbia River, preserving the environmental conditions and genetic variation that maintain these life history suites is a goal of conservation (Hoelzel et al., 2019).

Considerable progress has been made in identifying genomic regions associated with heritable life history variations, including age‐at‐maturity in anadromous steelhead. Recently, Waters et al. (2021) discovered through genome‐wide association analyses and Willis et al. (2020) confirmed via mixed‐effects association modeling that a region of chromosome 25 abutting the gene “sine oculis homeobox homolog 6” (*SIX6*) was strongly associated with age‐at‐maturity. Curiously, however, the penetrance of alleles associated with younger age‐at‐maturity (shorter ocean duration) was considerably stronger in males than females, an observation also made for age‐at‐maturity associated genes in Atlantic salmon (*Salmo salar*), another iteroparous species (Barson et al., 2015). Moreover, size (fork length) showed a stronger association with these genes than ocean age, another pattern repeated in Atlantic salmon (Barson et al., 2015). This may suggest that the effect of this region on age‐at‐maturity is through metabolic or physiological processes and results in a threshold effect for when to migrate and mature.

In contrast, identifying the conditions influencing the proclivity to initiate spawning cycles in consecutive years in steelhead, as well as genetic factors that may influence individual thresholds to those conditions, has been challenging because of the complex nature of iteroparity and difficulty clarifying the relationship with physiological condition in the period leading up to and following maiden spawning (Birnie‐Gauvin et al., 2023; Pierce et al., 2017). Thorpe (1986) proposed a model for salmonids in which energetic status (energetic reserves or their rate of change, i.e., energetic balance) must remain sufficient during some critical period to initiate a spawning cycle, and this critical period is hypothesized to occur during the year preceding spawning (see also Thorpe, 2007; Thorpe et al., 1998). For inland lineage Columbia basin steelhead, energy reserves preceding spawning are strongly challenged by 5–10 months of fasting during upstream migration to distant interior destinations (Keefer et al., 2008, 2018; Penney & Moffitt, 2014). Post‐spawning survival of steelhead (kelts) brought into the hatchery for reconditioning appears to be strongly related to the general health and physiological condition of the fish (average survival, 38%; Hatch et al., 2013). However, the magnitude of energy reserves (e.g., muscle lipid store or Fulton's condition factor) at or soon after maiden spawning showed little correlation with later rematuration for consecutive spawning (Jenkins et al., 2019, 2020, 2023). Jenkins et al. (2023) discovered that energy balance (the rate of change of energy reserves) at spawning strongly influenced the proclivity of inland steelhead to spawn again, since fish with positive energy balance rematured at higher rates in both fed and fasted treatments. However, feeding rather than fasting in a 10 week window following spawning increased the occurrence of consecutive versus skip spawning in female steelhead that were in negative energy balance at maiden spawning. This implies that the critical processes determining the ability to remature the following season includes energetic status prior to maiden spawning as well as resource availability during the immediate post‐spawn period (Jenkins et al., 2023; Pierce et al., 2017). However, since many females did not remature despite post‐spawn feeding and similarity in condition assessments at spawning (35%–75% rematuration; Pierce et al., 2017; Jenkins et al., 2020, 2023), it appears that individual energetic status thresholds differ, and so genetic factors influencing this threshold and the ability to mobilize and direct energy flows to initiate a subsequent reproductive cycle likely also play an important role.

Discovery of genetic markers that influence the proclivity to initiate consecutive spawning cycles would enhance knowledge of this complex maturation process, but also offer resources for kelt reconditioning efforts. Multiple programs have been developed that recondition (feed and medicate) post‐spawn steelhead, releasing them the following season with incoming spawners to boost natural spawning steelhead abundance for ESA listed populations. Our objective in this study was to identify regions of the genome associated with the phenology of iteroparity (consecutive or skip spawning) using low coverage whole genomic sequencing to reveal genetic markers that may be used for conservation and recovery of steelhead. To do this, we utilized low coverage whole genomic sequencing from two pairs of consecutive and skip spawning steelhead females from kelt reconditioning programs in the Columbia Basin. In addition, a second whole genomic sequencing dataset was examined to identify if regions of the genome associated with the phenology of iteroparity overlap with those associated with age‐at‐maturity in steelhead.

## 2 METHODS

### 2.1 Dataset 1: consecutive and skip spawning steelhead kelts

**FIGURE 1:**
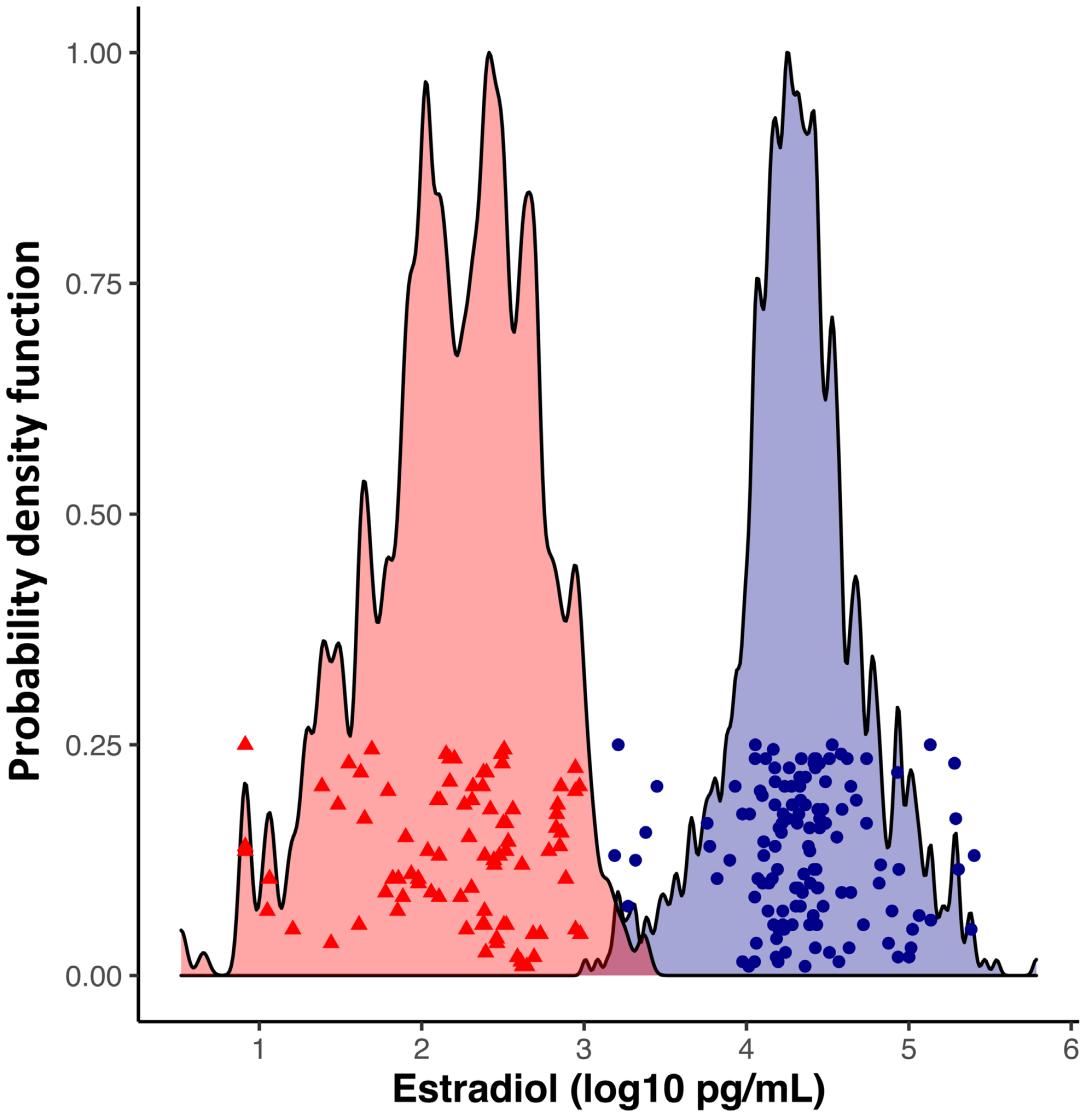
Log10 estradiol‐17β values for the individuals classified as consecutive (blue) or skip (red) spawning female steelhead (Dataset 1) and for which sequence data were collected (dots; y‐axis values arbitrary), and probability density function for all individuals classified but not analyzed from the same years (shaded area).

Samples consisted of two pairs of consecutive or skip spawning females reconditioned at two facilities in the interior Columbia River Basin, with spawning phenotype determined by plasma estradiol‐17β assay after 20 weeks of reconditioning (Figure 1) (Hatch et al., 2013; Jenkins et al., 2019, 2020, 2023; Pierce et al., 2017). The sample pairs were otherwise distinct in location and phenotype (Table S1): one sample pair consisted of hatchery‐origin fish collected in 2013, 2015, and 2016 for broodstock as they returned to the Dworshak National Fish Hatchery (near Ahsahka, Idaho) on the North Fork of the Clearwater River and subsequently air‐spawned. Dworshak individuals were restricted to 4 year‐maiden age (2‐river, 2‐ocean) individuals to focus on the predominant age class in this population. The other sample consisted of wild‐origin, natural spawning kelts outmigrating in the Yakima River and collected from the Chandler Juvenile Monitoring Facility at Prosser Dam (Prosser, WA) in 2015, 2016, 2018, and 2019. These Yakima individuals had unknown and presumably mixed ages reflecting the skew of Yakima fish toward 1‐ocean age‐at‐maturity (Hess et al., 2023). These two tributaries are genetically distinct and treated as different steelhead reporting groups, though they are part of the same Columbia River inland steelhead lineage (Hess et al., 2016). The number of individuals per sample set depended on collection location, Dworshak (33 consecutive and 35 skip spawners) or Yakima (56 consecutive and 59 skip spawners). Libraries were created for low coverage whole genome sequencing from genomic DNA extracted using Chelex (Bio‐Rad). Library preparation followed the individual barcoding protocol from Horn et al. (2020). Briefly, DNA from multiple individuals was normalized and used in a modified version of the Illumina Nextera protocol using indexed primers for each individual fish. Indexed DNA from each individual was pooled equimolarly in locality‐phenotype groups and sequenced paired‐end, 2 × 150 bp, on an Illumina NextSeq 550 in pairs by locality.

Sequence data were processed using the pipeline poolparty2 (Micheletti & Narum, 2018; Willis et al., 2023): quality‐trimmed reads (sliding window minimum quality of 20 with minimum retained length of 50 bp) were mapped to the rainbow trout genome assembly USDA Arlee 1.1 (GCA_013265735.3) with the ppalign module, applying a minimum mapQ ≥20, and sites with ≥10 reads and ≥3 individuals per locality‐x‐phenotype sample, global minor allele frequency ≥0.005, snpQ ≥20, and more than 3 bases away from an insertion–deletion event were retained. We note that the acrocentric chromosomes Omy30, Omy31, and Omy32 in the Arlee hatchery karyotype (*N* = 32) are homologous with the p‐arms of metacentric chromosomes Omy04, Omy14, and Omy25 in the more common (Swanson) *O. mykiss* karyotype (*N* = 29), and our notation below reflects that homology (e.g., “Omy30_Omy04p” is Arlee chromosome 30, which corresponds to the p‐arm of Swanson chromosome 4)(Gao et al., 2021; Pearse et al., 2019). Retained sites from ppalign were analyzed using the ppanalyze module and associated utilities of poolparty2 by calculating mean F_ST_ between spawning phenotypes, Fisher's Exact test (FET) of allele proportions between phenotypes, and Cochran–Mantel–Haenszel test (CMH) of replicate allelic differences between phenotype pairs (Cochran, 1954; Mantel & Haenszel, 1959). These raw results were further processed with site‐aggregate methods: F_ST_ was summarized in sliding windows of 100Kbp in steps of 5Kbp, while FET and CMH results were analyzed with local score smoothing (Fariello et al., 2017), with the 75th, 85th, 95th, and 99th quantiles of log10 p‐values utilized as the smoothing parameter, ξ. The ξ values represent a tradeoff in power and precision, with lower values (less smoothing) reflecting more power and higher values (more smoothing) reflecting greater precision in defining outlier regions (Fariello et al., 2017). We highlight outlier regions identified consistently by local score with multiple quantiles of ξ for both CMH and FET values, including local score analysis of FET performed for each of the localities separately.

We used angsd (Korneliussen et al., 2014) to generate genotype likelihoods for each individual as input to calculate linkage disequilibrium among sites. Because the calculation of linkage and summarization into windows is computationally intensive, we only performed linkage calculation for chromosomes with outlier regions identified by local score as described above. As input to angsd, we utilized the filtered BAM files created by the ppalign module of poolparty2 as well as the filtered set of SNP variants. We applied filters similar to those utilized with ppanalyze, minimum of 10 reads and 3 individuals in each of 4 locality‐x‐phenotype samples, but also applied a minor allele frequency filter of 0.05 to reduce the number of sites with poor linkage information content. We subset the genotype likelihood files produced for the selected chromosomes and used this as input for ngsld (Fox et al., 2019), specifying to calculate LD among sites within 100Kbp (1Mbp for Omy25). We then summarized LD across each chromosome by calculating mean LD in sliding windows of 100Kbp in steps of 5Kbp using custom code in R, and identified significant outlier regions as series of ≥20 window‐steps (with gaps of 2 or fewer between outlier window‐steps) with mean LD greater than 2x interquartile range (IQR) after excluding high background LD SNPs around the centromere of each chromosome. Although requiring any number of series of outlier regions may be effective, we found that minima of 10–20 sets a conservative boundary, depending on background differentiation (Willis et al., 2023). For the region associated with age‐at‐maturity on Omy25, we also tested for long‐distance linkage disequilibrium, that is, for non‐adjacent genomic regions which show linkage disequilibrium that putatively derives from processes other than physical proximity (e.g., selection on a phenotype affected by both regions). To do this, we calculated mean LD among sites in sliding windows ranging from 300Kbp to 1Mbp, in 5Kbp steps, as well as the mean LD among sites in the 100Kbp at both ends of these windows (minimum gap among sites: window width minus 2x100Kbp). We then tested whether mean LD was greater at the ends than over the whole region for each window size, using Wilcoxon signed rank tests for groups of 20 steps (@5Kbp each for 100Kbp total). Because of the computational demand of calculating LD and its summaries, we only estimated long range LD in 15Mbp of Omy25 centered on the age‐at‐maturity‐associated region. We provide the code used to execute the angsd and ngsld runs (Supplemental File S1).

### 2.2 Dataset 2: male and female steelhead of variable ocean age

We reanalyzed data from six pooled DNA samples (NCBI PRJNA650380) used in Waters et al. (2021) consisting of a pair of 1‐ocean and 2‐ocean age‐at‐maturity males from each of three Columbia Basin hatcheries (Dworshak FH, DWO; Pahsimeroi FH, PAH; Wallowa FH, WAL), mapping them to a more recent rainbow trout genome (Arlee 1.1 GCA_013265735.3, as above). We also analyzed a pair of pooled samples from females from one of the same hatcheries (Pahsimeroi), and a sample of 1‐ocean or 2‐ocean females (but not both) from two of the other hatcheries; these were prepared and sequenced concurrently to those presented in the Waters et al. (2021) study but not previously analyzed. Ocean age for these individuals was calculated from scales and confirmed with parentage‐based tagging. The number of individuals per pooled library ranged from 27 to 90 (mean 47). Data were processed similarly as above with poolparty2, including discarding reads trimmed below 50 bp with a sliding quality of ≥20, and filtering SNPs if they were below a PHRED quality of 20, three or fewer bases from an insertion–deletion position, observed in fewer than 10 reads in each sample pool, or were below a global minor allele frequency of 0.005. Because these data were pooled without barcoding individual DNA samples, we were unable to assess number of individuals per sample observed at a site, or normalize the data, as for Dataset 1. We similarly analyzed retained sites by assessing genetic divergence among samples (F_ST_) in sliding windows of 100Kbp in 5kbp steps and performing local score analyses of FET and CMH significance values across four quantiles of significance values as ξ. We ran FET analyses for all samples, male and female samples separately, and the female pair from Pahsimeroi, and ran CMH for all paired samples and the male pairs separately. For the top 10 SNPs (by CMH significance) in each outlier region associated with either age‐at‐maturity or the phenology of iteroparity, we compared allele frequencies between males and females of different ages and consecutive vs. skip spawning females, hypothesizing that differences among females of one phenotype would imply differences in the other phenotype. We note that we were unable to assess linkage disequilibrium in these samples because of their pooled nature.

## 3 RESULTS

### 3.1 Genomic regions associated with the phenology of iteroparity (dataset 1)

**FIGURE 2:**
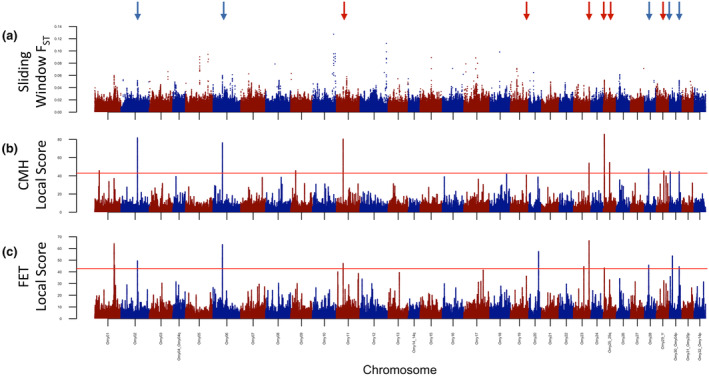
Site‐aggregate results from the analysis of low coverage whole genome sequencing from consecutive and skip spawning female steelhead (Dataset 1) with poolparty2. (a) Sliding window F_ST_ in windows of 100Kbp and 5Kbp steps. (b) Local score analysis of Fisher's Exact Test using ξ value representing the 85th quantiles of significance values. (c) Local score analysis of the Cochran–Mantel–Haenszel Test using ξ value representing the 85th quantiles of significance values (red lines: mean FDR 0.05). Arrows in each figure represent genomic regions identified in multiple tests associated with iteroparous spawning phenology.

Trimmed, mapped, and filtered reads of 298–411 million per sample resulted in 67.9%–71.1% of the genome covered at a minimum of 10 reads per sample, with a median coverage per individual of 0.48 (0–1.79) at variant sites for Dataset 1 (Figure S1). The ppalign module identified 12,114,378 variants after global filters, and when sample‐specific filters were applied in ppanalyze, 10,214,054 variants were retained for analysis. The raw results from F_ST_, FET, and CMH analyses revealed many individual outlier sites, but few obvious regions of strong association (Figure S2). F_ST_ summarized in sliding windows identified more obvious outlier regions (Figure 2), and some of these were also identified in local score analyses of FET and CMH results (Figure 2, Figure S3, Supplemental File S2). We identified 11 regions highlighted by local score with two or more quantiles of ξ for both CMH and FET values, on chromosomes Omy02, Omy06, Omy11, Omy19, Omy23, Omy25q (two regions), Omy28, Omy29_XY, and Omy30_Omy04p (two regions). Most of these outlier regions overlapped with genic regions or were transcriptionally upstream from adjacent genes (Figure S4, Supplemental File S2). Notably, the outlier region on Omy28 and one of the regions on Omy30 were next to or overlapped a duplication of three genes with a preserved synteny: two insulin‐like growth factor‐binding proteins (*IGFBP1* and *IGFBP3*) and tensin 3 (*TNS3*), as annotated. The outlier region was positioned differently relative to these genes on each chromosome and each was recovered in one or the other of the separate analyses of each locality as well as together (Supplemental File S2), suggesting outlier status was not a false positive derived from the duplication itself.

Of the 12,114,378 variant sites that poolparty2 identified with global filters in Dataset 1, angsd corroborated 12,108,129 of these after applying its own read and variant filters. Of these, only 9,499,160 sites were retained from population‐specific filters (minimum reads and individuals) and 7,968,617 sites were retained with a global MAF of ≥0.05. Using 2xIQR of LD to identify outliers, several to many regions were identified as outliers on each chromosome (Figure S4). Two of these LD outlier regions, on Omy25q and Omy29_XY corresponded to regions deemed significant by local score tests, while another local score‐significant region on Omy25q was an LD outlier but lay in the centromeric region (Figure S4).

**FIGURE 3:**
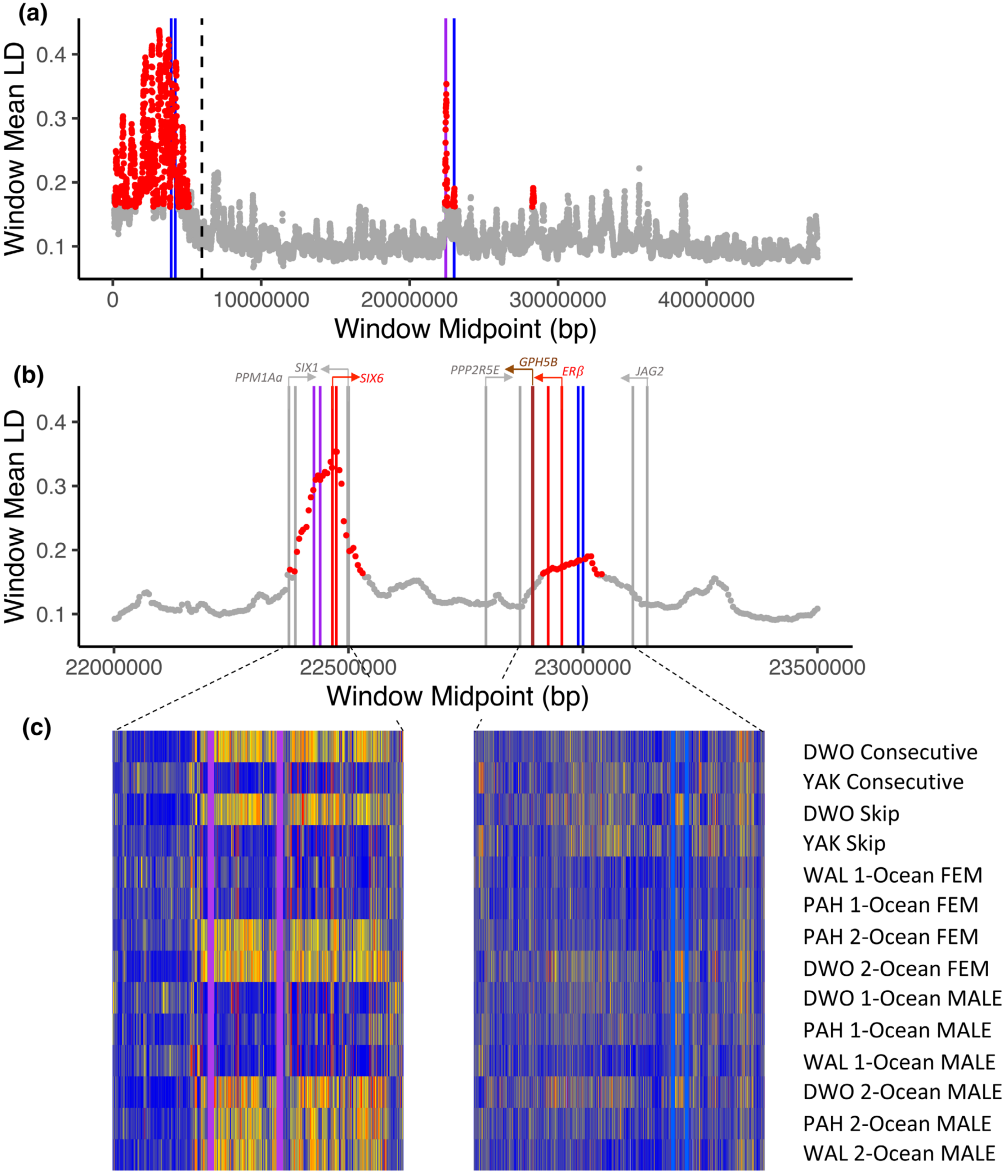
Mean linkage disequilibrium among sites on Omy25 q‐arm in sliding windows of 100Kbp and steps of 5Kbp, with LD outlier windows (red) identified as ≥2xIQR after excluding the centromeric region (bounds indicated by dashed line) in consecutive and skip spawning female steelhead (Dataset 1). Regions significant in local score analyses of consecutive and skip spawning female steelhead are indicated with blue lines, and the region significant in variable ocean age female steelhead is indicated with purple lines. (a) Full extent of Omy25 (b) 22–23.5 Mbp, the area containing the *SIX6* and *ERß/GPHB5* outlier regions. Genes overlapping or transcriptionally downstream of the outlier regions are indicated with red or brown lines, and other adjacent genes are identified with grey lines. The annotated direction of transcription is indicated with arrows above each gene; for full annotation, see Supplemental File S2. (c) Heat maps indicating differences in allele frequency for the outlier regions of Omy25 among consecutive and skip spawning and variable age‐at‐maturity female steelhead. The y‐axis lists the collection based on location, sex, and phenotype (Table S1).

On chromosome Omy25q, one local score and LD outlier region (22.99–23.06 Mbp) lay adjacent (~600 Kbp) to the region previously associated with age‐at‐maturity (22.43 Mbp), that is, the *SIX6* gene and the intergenic region upstream of it, which also exhibited LD but not local score outlier status in this dataset (Figure 3). This newly identified region reflected the intergenic region immediately upstream of two genes (3′ of genes transcribed in reverse on this assembly): the estrogen receptor *β* (*NR3A2* or *ER*β) and a homolog of glycoprotein hormone beta‐5 (*GPHB5*). The orientation of these genes suggests the highlighted region may contain an upstream regulatory region for one or both genes, whereas other proximal genes are oriented inconsistent with this. While both the age‐at‐maturity‐ and this iteroparity‐associated regions appear to reside in a broader region of higher LD on Omy25q, they exhibited distinct LD outlier status with respective local peaks of LD (Figure 3). While we did observe linkage between these two genomic regions, this appears to derive from the generally higher LD of the containing LD island rather than long‐distance LD per se, since LD of each region with the closer, intervening sites was always higher than between the regions themselves, and the mean of the complete window containing these sub‐regions was always higher than the LD between the 100Kbp on either end, regardless of window width (Figure S5).

### 3.2 Genomic regions associated with age at maturity across sexes (dataset 2)

**FIGURE 4:**
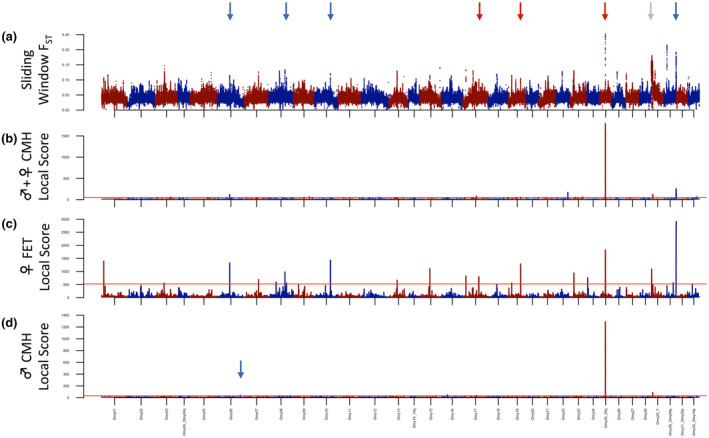
Site‐aggregate results from the analysis of low coverage whole genome sequencing from female and male steelhead of variable ocean age‐at‐maturity (Dataset 2) with poolparty2. (a) Sliding window F_ST_ in windows of 100Kbp and 5Kbp steps. (b,d) Local score analysis of the Cochran–Mantel–Haenszel Test using ξ values representing the 85th quantiles of significance values in females+males or males, respectively. (c) Local score analysis of Fisher's Exact Test using ξ value representing the 85th quantiles of significance values in females (red lines: mean FDR 0.05). Arrows in each figure represent genomic regions identified in multiple tests associated with variable ocean age‐at‐maturity; for annotation, see Supplemental File S3.

After trimming, mapping, and quality filtering using the ppalign module, mapped reads per sample ranged from 215 to 471 million, and ppstats revealed that this resulted in between 50%–71% of the genome being covered at a minimum of 10 reads in each sample for Dataset 2 (Figure S6). ppalign initially identified 20,637,227 variants with global filters, which was reduced to between 9,023,073 to 12,963,332 variants after sample‐specific minima were applied in ppanalyze, depending on the samples analyzed. The sliding windows of genetic divergence and local score analyses revealed a strong region of divergence on chr. 25 containing the *SIX6* gene (Figure 4, Figures [Link], [Link], Supplemental File S3), as noted by Waters et al. (2021). While the male local score analyses emphasized only the Omy25q (*SIX6*) region and another, much less significant region on Omy06 (Figure S9), the female analyses highlighted a number of other regions, including a different region on Omy06, as well as regions on Omy08, Omy10, Omy17, Omy19, and Omy30_Omy04p (Figure 4; Figure S8). Both sexes also showed outlier regions bordering the sex‐determining on the Y (*SDY*) gene on Omy29_XY, which is probably attributable to reduced recombination. Notably, neither of the homologs of transcription cofactor vestigial‐like protein 3 (*VGLL3*) on Omy03 or Omy22, a gene strongly associated with age‐at‐maturity in Atlantic salmon, appeared in any of these analyses. None of the outlier regions identified by analysis of variable ocean age individuals overlapped with those identified as associated with the phenology of iteroparity, apart from the proximity of the adjacent regions on Omy25q. Similarly, when we examined allele frequencies of the ten variants with the highest CMH scores in each of the iteroparity‐associated regions in the variable ocean age individuals, we saw no clear pattern except for the *ERB*/*GPHB5* region on Omy25: for these sites, we saw strong differences between females of different ages, but not males (Figure 3, Supplemental File S2). We did not see the converse pattern, however, consecutive and skip spawning females did not show differences in the top‐scoring sites for the age‐at‐maturity‐associated region near *SIX6*. Rather, differences among these consecutive and skip spawning fish reflected the strong differences in age‐at‐maturity proportions between the two localities.

## 4 DISCUSSION

Recent years have seen considerable effort and notable success using allele frequencies derived from low coverage whole genome sequencing data (lcWGS) to identify loci of large effect which are strongly associated with heritable traits, including life history variations important for the conservation of salmonid species (e.g., Waples et al., 2022). In many of these landmark studies, the large effect loci were apparent in most or all of the presented analyses and exhibited significance or measures of association orders of magnitude exceeding the rest of the genome, such as that seen with the *GREB1L*‐*ROCK1* region and its association with run timing (e.g., Narum et al., 2018, 2023).

In contrast, the genomic regions we infer as being associated with the phenology of iteroparity, or as mediating age‐at‐maturity within sex in steelhead, were identified in several but not all of our analyses and often exhibited lower magnitudes of association. Also, these magnitudes were similar to regions which only appeared in one or a few analyses and which we inferred to only be spuriously associated with these phenotypes. While this may provide less confidence than studies with single regions of major effect, several things bear consideration when interpreting results of this nature. Genome‐wide association analyses using allele frequencies and categorical variables rather than individual‐level genotype and phenotype data depend on two things: discrete, heritable phenotypic states and strong effects from relatively few genes. As the additive variance is distributed across more genes, the combinatorial effects of frequencies at different alleles requires the power of detection to increase proportionately. Conversely, power is lost as the degree of additive genetic variance recorded in the response (phenotypic) states is reduced because of less direct effects of the locus on the phenotype. Traits like anadromy vs. residency or consecutive vs. skip spawning may prima facie appear distinct, but are likely strongly condition and context‐dependent (e.g., Collins et al., 2022; Jenkins et al., 2023; Kendall et al., 2015), meaning that they are in fact integrated phenotypes reflecting a number of related processes, including metabolism, growth trajectories, immunity, etc., the many genes of large and small effects that contribute to those process, and the compounding of stochastic environmental effects on each. Thus, detection of polygenic association with complex phenotypes is expected to be challenging, and the associations are likely to be of relatively small effect. However, designs for future studies could be enhanced by incorporating extensive individual‐level data on the many physiological processes and environmental conditions that likely affect the final complex phenotype, as well as high coverage sequencing to genotype large sample sizes (Lou et al., 2021).

Nonetheless, we report several genomic regions which have repeated associations with this complex phenotype, proclivity to remature and spawn consecutively. These results were supported through inclusion of estimates of linkage disequilibrium by integration of the poolparty2 and angsd bioinformatic suites. poolparty2 and angsd have distinct though overlapping functionalities, including the conveyance of unmapped reads through quality trimming, mapping, quality assurance, and group‐level association analyses (poolparty2) and the estimation of genotype likelihoods and individual‐level association analyses (angsd). While it was shown recently that poolparty2 will provide more reliable variant identification and allele frequency estimation than angsd in some cases (Willis et al., 2023), the complement of these two suites facilitates a number of analyses such as ngsld that were critical for inference of linkage among the recovered SNPs in this study. Specifically, we used the observation of linkage disequilibrium to further investigate regions associated with our focal phenotypes. High LD is expected when nearby variants “hitchhike” with mutations under strong selection or when multiple causal mutations with similar effects are co‐selected such that alternative haplotypes are created (Nielsen et al., 2005). However, LD can also occur due to background processes, including historical population structure (e.g., Wahlund effects) and segregating structural variants (e.g., inversions) that create regions of low recombination unrelated to selection. Thus we have not highlighted regions that show high LD but were not significant in analyses to identify repeated divergence among phenotype replicates (FET or CMH).

Of the several regions we report, the one that appears the most intriguing is that on Omy25q adjacent to the age‐at‐maturity associated region, with which it shares an island of LD. The genes near this region on Omy25q have not been previously associated with iteroparity or age‐at‐maturity to our knowledge, but there is a clear link to their possible influence on spawning phenology. In humans, *ER*β, an estradiol‐sensitive transcription regulator primarily expressed in the uterus and other reproductive tissues and showing an expression correlated with menstruation (Matsuzaki et al., 1999), may inhibit cell proliferation and oppose the actions of the alpha receptor (*ER*α) (Paech et al., 1997; Weihua et al., 2000). In iteroparous fishes including *O. mykiss*, signaling through *ER*β has been proposed to prime the liver, the primary site of vitellogenesis, to respond to estradiol early in gonadal recrudescence (Nelson & Habibi, 2013). Estradiol‐17β, the circulating estrogen whose pattens of expression immediately following spawning shows inconsistent association with consecutive versus skip spawning, is nonetheless a clear marker of rematuration in consecutive spawners by 20 weeks post‐spawn (Jenkins et al., 2020, 2023). Variation in the expression of *ER*β or its sensitivity to estradiol could theoretically moderate the proclivity of individuals to remature immediately or delay rematuration. The other gene adjacent to the CMH‐significant region, lying 32.5Kbp from the CDS for *ER*β, *GPHB5* codes for a glycoprotein which forms heterodimers with another glycoprotein, *GPHA2*, to become the hormone thyrostimulin (Nakabayashi et al., 2002). Thyrostimulin is an analog of the pituitary‐produced thyroid stimulating hormone (TSH), which activates the thyroid stimulating hormone receptor (TSHR) as part of the hypothalamus‐pituitary‐thyroid axis to regulate metabolism and growth through thyroxin production in the thyroid. Thyrostimulin has an equal to superior binding affinity for TSHR, but unlike TSH, in mammals co‐expression of *GPHB2* and *TSHR* appears to be highest in the ovaries rather than the pituitary (Nagasaki et al., 2006; Sun et al., 2010), where it is also sensitive to estradiol and appears to act in paracrine fashion to regulate cell proliferation (Huang et al., 2016; Sun et al., 2010). Thus, *GPHB5* is also a plausible regulator of proclivity to spawn consecutively through the coordination of metabolism and gonadal maturation. Notably, Willis et al. (2020) observed that the penetrance of alleles in the *SIX6* region promoting early maturation and younger age‐at‐maturity was higher in males than females, and speculated about another sex‐linked or sex‐dependent locus mediating the effect of that genomic region (see also Barson et al., 2015). Given the report of these additional genes' sensitivity to estrogen, expression in the reproductive tissues, and putative role in regulation of cell growth and metabolism, it is conceivable that these may act both to moderate the effect of *SIX6* on age‐at‐maturity and influence the phenology of iteroparity.

The results from the examination of males and females of different ocean ages (ages‐at‐maturity) corroborate the more complex architecture of age‐at‐maturity in female steelhead as well. Unlike males, which show a clear association primarily with the *SIX6* gene region on Omy25, females show significant associations with a number of other genomic regions, any of which could hypothetically moderate the effects of the *SIX6* region on spawning phenology. Moreover, even though none of these primarily female‐associated genomic regions were shared between analyses, the observation that females but not males show strong differences in the *ER*β/*GPHB5* region of Omy25 suggests that these genomic regions may influence both traits in females. These genes, *SIX6*, *ER*β, and *GPHB5*, occupy an island of LD on Omy25, which may help coordinate effects on spawning phenology during the months preceding migration into freshwater (Jenkins et al., 2023). During this time, individual thresholds to energetic status likely influence whether steelhead return to spawn in the current season or delay one or more additional years (age‐at‐maturity), as well as whether and when they will have the energetic reserves to spawn again (consecutive vs. skip spawn). Moreover, as observed by Barson et al. (2015), the effects of *SIX6*, a transcription factor affecting the hypothalamus–pituitary–gonadal axis and associated with size and age at maturity in humans, may be through size at age via growth or resource partitioning for maturation rather than through a direct effect on maturation timing. Atlantic salmon also show an additional strong association of age‐at‐maturity with the *VGLL3* gene that is not apparent in steelhead (Barson et al., 2015; Waters et al., 2021 and the present study), but Aykanat et al. (2019) observed that Atlantic salmon with *VGLL3* alleles promoting early maturation (shorter ocean duration and younger age‐at‐maturity) were much more likely to be iteroparous than fish with alleles associated with older maturation. This relationship between age at maturity and iteroparity has been observed in multiple studies of steelhead (Keefer et al., 2008, 2018; Narum et al., 2008; Wertheimer & Evans, 2005). Thus, it stands to reason that two condition‐dependent reproductive traits whose sensitive period appears to overlap and exhibit correlated fitness would have a similar genetic basis.

Several other regions identified here in association with spawning phenology in female steelhead remain to be explored, including the association of the phenology of iteroparity with duplicated regions containing insulin‐like growth factor‐binding proteins, which may enhance or attenuate the effects of the growth hormone/insulin‐like growth factor endocrine system on energy balance and whole‐body or tissue‐specific cell proliferation via their binding of insulin‐like growth factors, depending on context (Sheridan, 2021). Clarifying the specific targets of association (selection) within these regions and their action in mediating spawning phenology will require considerable effort.

## 5 CONCLUSIONS

Using allele frequencies of consecutive and skip spawning female Columbia River steelhead from two populations derived from low coverage whole genome sequencing, we identified genomic regions on several chromosomes which repeatedly showed association with the phenology of iteroparity. One of these genomic regions of association localized to the q‐arm of chromosome 25, which is adjacent to a region that has previously been associated with age‐at‐maturity in steelhead. This adjacent region of Omy25 contained two estrogen‐sensitive genes, *ER*β and *GPHB5*, that are expressed in and moderate cellular processes of the reproductive tissues. These two genes may have interacting effects on age‐at‐maturity and proclivity to remature the following reason, which increasingly appear to be overlapping if not related processes. However, further work is needed to validate and investigate the utility of variants this *ER*β/*GPHB5* region to predict consecutive versus skip spawning phenology as well as the expression of age‐at‐maturity in steelhead, along with the numerous other genomic regions that may mediate the expression of these traits in females versus males.

## Supporting information


Figure S1.

Figure S2.

Figure S3.

Figure S4.

Figure S5.

Figure S6.

Figure S7.

Figure S8.

Figure S9.

Table S1.

File S1.

File S2.

File S3.

Data S1.

## Data Availability

Data referenced in this manuscript are available from NBCI Short Read Archive (PRJNA870412, PRJNA650380). The pipeline used for bioinformatic processing of these data, PoolParty2, is available from Github (https://github.com/stuartwillis/poolparty). Additional code is available as Supplemental File S1.

## References

[eva13622-bib-0001] Aykanat, T. , Ozerov, M. , Vähä, J. P. , Orell, P. , Niemelä, E. , Erkinaro, J. , & Primmer, C. R. (2019). Co‐inheritance of sea age at maturity and iteroparity in the Atlantic salmon vgll3 genomic region. Journal of Evolutionary Biology, 32, 343–355. 10.1111/jeb.13418 30697850

[eva13622-bib-0002] Barson, N. J. , Aykanat, T. , Hindar, K. , Baranski, M. , Bolstad, G. H. , Fiske, P. , Jacq, C. , Jensen, A. J. , Johnston, S. E. , Karlsson, S. , Kent, M. , Moen, T. , Niemelä, E. , Nome, T. , Næsje, T. F. , Orell, P. , Romakkaniemi, A. , Sægrov, H. , Urdal, K. , … Primmer, C. R. (2015). Sex‐dependent dominance at a single locus maintains variation in age at maturity in salmon. Nature, 528, 405–408. 26536110 10.1038/nature16062

[eva13622-bib-0003] Berg, O. K. , Thronæs, E. , & Bremset, G. (1998). Energetics and survival of virgin and repeat spawning brown trout (Salmo trutta). Canadian Journal of Fisheries and Aquatic Sciences, 55, 47–53.

[eva13622-bib-0004] Birnie‐Gauvin, K. , Bordeleau, X. , Eldøy, S. H. , Bøe, K. , Kristensen, M. L. , Nilsen, C. I. , & Lennox, R. J. (2023). A review of iteroparity in anadromous salmonids: Biology, threats and implications. Reviews in Fish Biology and Fisheries, 33(4), 1–21.

[eva13622-bib-0005] Bowersox, B. J. , Corsi, M. P. , McCormick, J. L. , Copeland, T. , & Campbell, M. R. (2019). Examining life history shifts and genetic composition in a hatchery steelhead population, with implications for fishery and ocean selection. Transactions of the American Fisheries Society, 148, 1056–1068. 10.1002/tafs.10199

[eva13622-bib-0006] Busby, P. , Wainwright, T. , & Bryant, G. (2000). Status review of steelhead from Washington, Idaho, Oregon, and California. In E. E. Knudsen & D. M. McDonald (Eds.), Sustainable Fishery Management, pp. 173–175.

[eva13622-bib-0007] Carlson, S. M. , & Seamons, T. R. (2008). SYNTHESIS: A review of quantitative genetic components of fitness in salmonids: Implications for adaptation to future change. Evolutionary Applications, 1, 222–238. 25567628 10.1111/j.1752-4571.2008.00025.x PMC3352437

[eva13622-bib-0008] Christie, M. R. , McNickle, G. G. , French, R. A. , & Blouin, M. S. (2018). Life history variation is maintained by fitness tradeoffs and negative frequency‐dependent selection. Proceedings of the National Academy of Sciences of the United States of America, 115(17), 4441–4446. 29643072 10.1073/pnas.1801779115 PMC5924930

[eva13622-bib-0009] Cochran, W. G. (1954). Some methods for strengthening the common *χ* ^2^ tests. Biometrics, 10, 417.

[eva13622-bib-0010] Collins, E. E. , Romero, N. , Zendt, J. S. , & Narum, S. R. (2022). Whole‐genome resequencing to evaluate life history variation in anadromous migration of Oncorhynchus mykiss. Frontiers in Genetics, 13, 795850. 35368705 10.3389/fgene.2022.795850 PMC8964970

[eva13622-bib-0011] Copeland, T. , Ackerman, M. W. , Wright, K. K. , & Byrne, A. (2017). Life history diversity of snake river steelhead populations between and within management categories. North American Journal of Fisheries Management, 37, 395–404.

[eva13622-bib-0012] Fariello, M. I. , Boitard, S. , Mercier, S. , Robelin, D. , Faraut, T. , Arnould, C. , Recoquillay, J. , Bouchez, O. , Salin, G. , Dehais, P. , Gourichon, D. , Leroux, S. , Pitel, F. , Leterrier, C. , & SanCristobal, M. (2017). Accounting for linkage disequilibrium in genome scans for selection without individual genotypes: The local score approach. Molecular Ecology, 26(14), 3700–3714. 28394503 10.1111/mec.14141

[eva13622-bib-0013] Fox, E. A. , Wright, A. E. , Fumagalli, M. , & Vieira, F. G. (2019). NgsLD: Evaluating linkage disequilibrium using genotype likelihoods. Bioinformatics, 35, 3855–3856. 30903149 10.1093/bioinformatics/btz200

[eva13622-bib-0014] Gao, G. , Magadan, S. , Waldbieser, G. C. , Youngblood, R. C. , Wheeler, P. A. , Scheffler, B. E. , Thorgaard, G. H. , & Palti, Y. (2021). A long reads‐based de‐novo assembly of the genome of the Arlee homozygous line reveals chromosomal rearrangements in rainbow trout. G3 Genes, Genomes, Genet, 11(4), jkab052. 10.1093/g3journal/jkab052 PMC8763230 33616628

[eva13622-bib-0015] Hatch, D. R. , Fast, D. E. , Bosch, W. J. , Blodgett, J. W. , Whiteaker, J. M. , Branstetter, R. , & Pierce, A. L. (2013). Survival and traits of reconditioned Kelt steelhead Oncorhynchus mykiss in the Yakima River, Washington. North American Journal of Fisheries Management, 33, 615–625.

[eva13622-bib-0016] Hess, J. E. , Ackerman, M. W. , Fryer, J. K. , Hasselman, D. J. , Steele, C. A. , Stephenson, J. J. , Whiteaker, J. M. , & Narum, S. R. (2016). Differential adult migration‐timing and stock‐specific abundance of steelhead in mixed stock assemblages. ICES. Journal of Marine Science and Application, 73, 2606–2615.

[eva13622-bib-0017] Hess, J. E. , Horn, R. L. , Stephenson, J. , Willis, S. C. , & Narum, S. R. 2023. Genetic assessment of Columbia River stocks, 1/1/2022–12/31/2022. Columbia River Inter‐Tribal Fish Comission.

[eva13622-bib-0018] Hoelzel, A. R. , Bruford, M. W. , & Fleischer, R. C. (2019). Conservation of adaptive potential and functional diversity. Conservation Genetics, 20, 1–5.

[eva13622-bib-0019] Horn, R. L. , Kamphaus, C. , Murdoch, K. , & Narum, S. R. (2020). Detecting genomic variation underlying phenotypic characteristics of reintroduced Coho salmon (Oncorhynchus kisutch). Conservation Genetics, 21(6), 1011–1021.

[eva13622-bib-0020] Huang, W. L. , Li, Z. , Lin, T. Y. , Wang, S. W. , Wu, F. J. , & Luo, C. W. (2016). Thyrostimulin‐TSHR signaling promotes the proliferation of NIH:OVCAR‐3 ovarian cancer cells via trans‐regulation of the EGFR pathway. Scientific Reports, 6, 27471. 27273257 10.1038/srep27471 PMC4895341

[eva13622-bib-0021] Jenkins, L. E. , Medeiros, L. R. , Graham, N. D. , Hoffman, B. M. , Cervantes, D. L. , Hatch, D. R. , Nagler, J. J. , & Pierce, A. L. (2023). Feeding after spawning and energy balance at spawning are associated with repeat spawning interval in steelhead trout. General and Comparative Endocrinology, 332, 114181. 36455641 10.1016/j.ygcen.2022.114181

[eva13622-bib-0022] Jenkins, L. E. , Pierce, A. L. , Caudill, C. C. , Graham, N. D. , Medeiros, L. R. , Hatch, D. R. , & Nagler, J. J. (2020). Effects of physiological condition on aspects of repeat spawning in female steelhead reconditioned in captivity. Transactions of the American Fisheries Society, 149, 213–224.

[eva13622-bib-0023] Jenkins, L. E. , Pierce, A. L. , Graham, N. D. , Medeiros, L. R. , Hatch, D. R. , & Nagler, J. J. (2019). Elevated plasma triglycerides and growth rate are early indicators of reproductive status in post‐spawning female steelhead trout (*Oncorhynchus mykiss*). Conservation Physiology, 7(1), coz038. 31380109 10.1093/conphys/coz038 PMC6659465

[eva13622-bib-0024] Keefer, M. L. , Jepson, M. A. , Clabough, T. S. , Johnson, E. L. , Narum, S. R. , Hess, J. E. , & Caudill, C. C. (2018). Sea‐to‐sea survival of late‐run adult steelhead (Oncorhynchus mykiss) from the Columbia and snake rivers. Canadian Journal of Fisheries and Aquatic Sciences, 75, 331–341.

[eva13622-bib-0025] Keefer, M. L. , Wertheimer, R. H. , Evans, A. F. , Boggs, C. T. , & Peery, C. A. (2008). Iteroparity in Columbia River summer‐run steelhead (Oncorhynchus mykiss): Implications for conservation. Canadian Journal of Fisheries and Aquatic Sciences, 65, 2592–2605.

[eva13622-bib-0026] Kendall, N. W. , McMillan, J. R. , Sloat, M. R. , Buehrens, T. W. , Quinn, T. P. , Pess, G. R. , Kuzishchin, K. V. , McClure, M. M. , & Zabel, R. W. (2015). Anadromy and residency in steelhead and rainbow trout (oncorhynchus mykiss): A review of the processes and patterns. Canadian Journal of Fisheries and Aquatic Sciences, 72, 319–342. 10.1139/cjfas-2014-0192

[eva13622-bib-0027] Korneliussen, T. S. , Albrechtsen, A. , & Nielsen, R. (2014). ANGSD: Analysis of next generation sequencing data. BMC Bioinformatics, 15, 356. 25420514 10.1186/s12859-014-0356-4 PMC4248462

[eva13622-bib-0028] Lou, R. N. , Jacobs, A. , Wilder, A. P. , & Therkildsen, N. O. (2021). A beginner's guide to low‐coverage whole genome sequencing for population genomics. P. In Molecular Ecology, 30(23), 5966–5993. 34250668 10.1111/mec.16077

[eva13622-bib-0029] Mantel, N. , & Haenszel, W. (1959). Statistical aspects of the analysis of data from retrospective studies of disease. Journal of the National Cancer Institute, 22(4), 719–748. 13655060

[eva13622-bib-0030] Matsuzaki, S. , Fukaya, T. , Suzuki, T. , Murakami, T. , Sasano, H. , & Yajima, A. (1999). Oestrogen receptor α and β mRNA expression in human endometrium throughout the menstrual cycle. Molecular Human Reproduction, 5(6), 559–564. 10341004 10.1093/molehr/5.6.559

[eva13622-bib-0031] Micheletti, S. J. , & Narum, S. R. (2018). Utility of pooled sequencing for association mapping in nonmodel organisms. Molecular Ecology Resources, 18, 825–837. 10.1111/1755-0998.12784 29633534

[eva13622-bib-0032] Moore, J. W. , Yeakel, J. D. , Peard, D. , Lough, J. , & Beere, M. (2014). Life‐history diversity and its importance to population stability and persistence of a migratory fish: Steelhead in two large north American watersheds. The Journal of Animal Ecology, 83, 1035–1046. 24673479 10.1111/1365-2656.12212

[eva13622-bib-0033] Nagasaki, H. , Wang, Z. , Jackson, V. R. , Lin, S. , Nothacker, H. P. , & Civelli, O. (2006). Differential expression of the thyrostimulin subunits, glycoprotein α2 and β5 in the rat pituitary. Journal of Molecular Endocrinology, 37(1), 39–50. 16901922 10.1677/jme.1.01932

[eva13622-bib-0034] Nakabayashi, K. , Matsumi, H. , Bhalla, A. , Bae, J. , Mosselman, S. , Hsu, S. Y. , & Hsueh, A. J. W. (2002). Thyrostimulin, a heterodimer of two new human glycoprotein hormone subunits, activates the thyroid‐stimulating hormone receptor. The Journal of Clinical Investigation, 109(11), 1445–1452. 12045258 10.1172/JCI14340 PMC150994

[eva13622-bib-0035] Narum, S. R. , Di Genova, A. , Micheletti, S. J. , & Maass, A. (2018). Genomic variation underlying complex life‐history traits revealed by genome sequencing in Chinook salmon. Proceedings of the Royal Society B: Biological Sciences, 285(1883), 1–9. 10.1098/rspb.2018.0935 PMC6083255 30051839

[eva13622-bib-0036] Narum, S. R. , Hatch, D. , Talbot, A. J. , Moran, P. , & Powell, M. S. (2008). Iteroparity in complex mating systems of steelhead Oncorhynchus mykiss (Walbaum). Journal of Fish Biology, 72, 45–60.

[eva13622-bib-0037] Narum, S. R. , Koch, I. J. , Horn, R. L. , Hess, J. E. , & Willis, S. C. (2023). Genetic variation associated with adult migration timing in lineages of Steelhead & Chinook Salmon in the Columbia River. Evolutionary Applications in review 10.1111/eva.13623. PMC10853649 38343781

[eva13622-bib-0038] Nelson, E. R. , & Habibi, H. R. (2013). Estrogen receptor function and regulation in fish and other vertebrates. General and Comparative Endocrinology, 192, 15–24. 23583769 10.1016/j.ygcen.2013.03.032

[eva13622-bib-0039] Nielsen, R. , Williamson, S. , Kim, Y. , Hubisz, M. J. , Clark, A. G. , & Bustamante, C. (2005). Genomic scans for selective sweeps using SNP data. Genome Research, 15, 1566–1575. 16251466 10.1101/gr.4252305 PMC1310644

[eva13622-bib-0040] Paech, K. , Webb, P. , Kuiper, G. G. , Nilsson, S. , Gustafsson, J. , Kushner, P. J. , & Scanlan, T. S. (1997). Differential ligand activation of estrogen receptors ERalpha and ERbeta at AP1 sites [see comments]. Science, 277(5331), 1508–1510. 9278514 10.1126/science.277.5331.1508

[eva13622-bib-0041] Pearse, D. E. , Barson, N. J. , Nome, T. , Gao, G. , Campbell, M. A. , Abadía‐Cardoso, A. , Anderson, E. C. , Rundio, D. E. , Williams, T. H. , Naish, K. A. , Moen, T. , Liu, S. , Kent, M. , Moser, M. , Minkley, D. R. , Rondeau, E. B. , Brieuc, M. S. O. , Sandve, S. R. , Miller, M. R. , … Lien, S. (2019). Sex‐dependent dominance maintains migration supergene in rainbow trout. Nature Ecology & Evolution, 3(12), 1731–1742. 31768021 10.1038/s41559-019-1044-6

[eva13622-bib-0042] Penney, Z. L. , & Moffitt, C. M. (2014). Proximate composition and energy density of stream‐maturing adult steelhead during upstream migration, sexual maturity, and Kelt emigration. Transactions of the American Fisheries Society, 143, 399–413.

[eva13622-bib-0043] Pierce, A. L. , Blodgett, J. W. , Cavileer, T. D. , Medeiros, L. R. , Boyce, J. , Caldwell, L. K. , Bosch, W. J. , Branstetter, R. , Fast, D. E. , Hatch, D. R. , & Nagler, J. J. (2017). Reproductive development in captive reconditioned female steelhead kelts: Evidence for consecutive and skip spawning life histories. Canadian Journal of Fisheries and Aquatic Sciences, 74, 1049–1060.

[eva13622-bib-0044] Quinn, T. P. , Seamons, T. R. , Vollestad, L. A. , & Duffy, E. (2011). Effects of growth and reproductive history on the egg size‐fecundity trade‐off in steelhead. Transactions of the American Fisheries Society, 140, 45–51. 10.1080/00028487.2010.550244

[eva13622-bib-0045] Schindler, D. E. , Armstrong, J. B. , & Reed, T. E. (2015). The portfolio concept in ecology and evolution. Frontiers in Ecology and the Environment, 13(5), 257–263.

[eva13622-bib-0046] Schindler, D. E. , Hilborn, R. , Chasco, B. , Boatright, C. P. , Quinn, T. P. , Rogers, L. A. , & Webster, M. S. (2010). Population diversity and the portfolio effect in an exploited species. Nature, 465, 609–612. 20520713 10.1038/nature09060

[eva13622-bib-0047] Sheridan, M. A. (2021). Coordinate regulation of feeding, metabolism, and growth: Perspectives from studies in fish. General and Comparative Endocrinology, 312, 113873. 34329604 10.1016/j.ygcen.2021.113873

[eva13622-bib-0048] Sun, S. C. , Hsu, P. J. , Wu, F. J. , Li, S. H. , Lu, C. H. , & Luo, C. W. (2010). Thyrostimulin, but not thyroid‐stimulating hormone (TSH), acts as a paracrine regulator to activate the TSH receptor in mammalian ovary. The Journal of Biological Chemistry, 285(6), 3758–3765. 19955180 10.1074/jbc.M109.066266 PMC2823517

[eva13622-bib-0049] Thorpe, J. E. (1986). Age at first maturity in Atlantic Salmon, Salmo salan freshwater period influences and conflicts with Smolting. In R. L. Saunders (Ed.), SALMONID AGE AT MATURITY, pp. 7–14.

[eva13622-bib-0050] Thorpe, J. E. (2007). Maturation responses of salmonids to changing developmental opportunities.

[eva13622-bib-0051] Thorpe, J. E. , Mangel, M. , Metcalfe, N. B. , & Huntingford, F. A. (1998). Modelling the proximate basis of salmonid life‐history variation, with application to Atlantic salmon, Salmo salar L. Evolutionary Ecology, 12, 581–599. 10.1023/A:1022351814644

[eva13622-bib-0052] Waples, R. S. , Ford, M. J. , Nichols, K. , Kardos, M. , Myers, J. , Thompson, T. Q. , Anderson, E. C. , Koch, I. J. , McKinney, G. , Miller, M. R. , Naish, K. , Narum, S. R. , O'Malley, K. G. , Pearse, D. E. , Pess, G. R. , Quinn, T. P. , Seamons, T. R. , Spidle, A. , Warheit, K. I. , & Willis, S. C. (2022). Implications of large‐effect loci for conservation: A review and case study with Pacific Salmon. The Journal of Heredity, 113, 121–144. 10.1093/jhered/esab069 35575083

[eva13622-bib-0053] Waters, C. D. , Clemento, A. , Aykanat, T. , Garza, J. C. , Naish, K. A. , Narum, S. , & Primmer, C. R. (2021). Heterogeneous genetic basis of age at maturity in salmonid fishes. Molecular Ecology, 30, 1435–1456. Blackwell Publishing Ltd. 33527498 10.1111/mec.15822

[eva13622-bib-0054] Weihua, Z. , Saji, S. , Mäkinen, S. , Cheng, G. , Jensen, E. V. , Warner, M. , & Gustafsson, J. Å. (2000). Estrogen receptor (ER) β, a modulator of ERα in the uterus. Proceedings of the National Academy of Sciences of the United States of America, 97(11), 5936–5941. 10823946 10.1073/pnas.97.11.5936 PMC18537

[eva13622-bib-0055] Wertheimer, R. H. , & Evans, A. F. (2005). Downstream passage of steelhead Kelts through hydroelectric dams on the lower Snake and Columbia Rivers. Transactions of the American Fisheries Society, 134(4), 853–865.

[eva13622-bib-0056] Willis, S. C. , Hess, J. E. , Fryer, J. K. , Whiteaker, J. M. , Brun, C. , Gerstenberger, R. , & Narum, S. R. (2020). Steelhead (Oncorhynchus mykiss) lineages and sexes show variable patterns of association of adult migration timing and age‐at‐maturity traits with two genomic regions. Evolutionary Applications, 13, 2836–2856. 33294026 10.1111/eva.13088 PMC7691471

[eva13622-bib-0057] Willis, S. C. , Micheletti, S. J. , Andrews, K. R. , & Narum, S. R. (2023). POOLPARTY2: An integrated pipeline for analyzing pooled or indexed low coverage whole genome sequencing data to discover the genetic basis of diversity. Molecular Ecology Resources Authorea June 06. 10.1111/1755-0998.13888 37921673

