# Supplementary figures and images for: A genomic region associated with iteroparous spawning phenology is linked with age‐at‐maturity in female steelhead trout
Source: Evol Appl. 2023 Dec 11;17(2):e13622. doi: 10.1111/eva.13622 (PMC10853659; doi:10.1111/eva.13622)

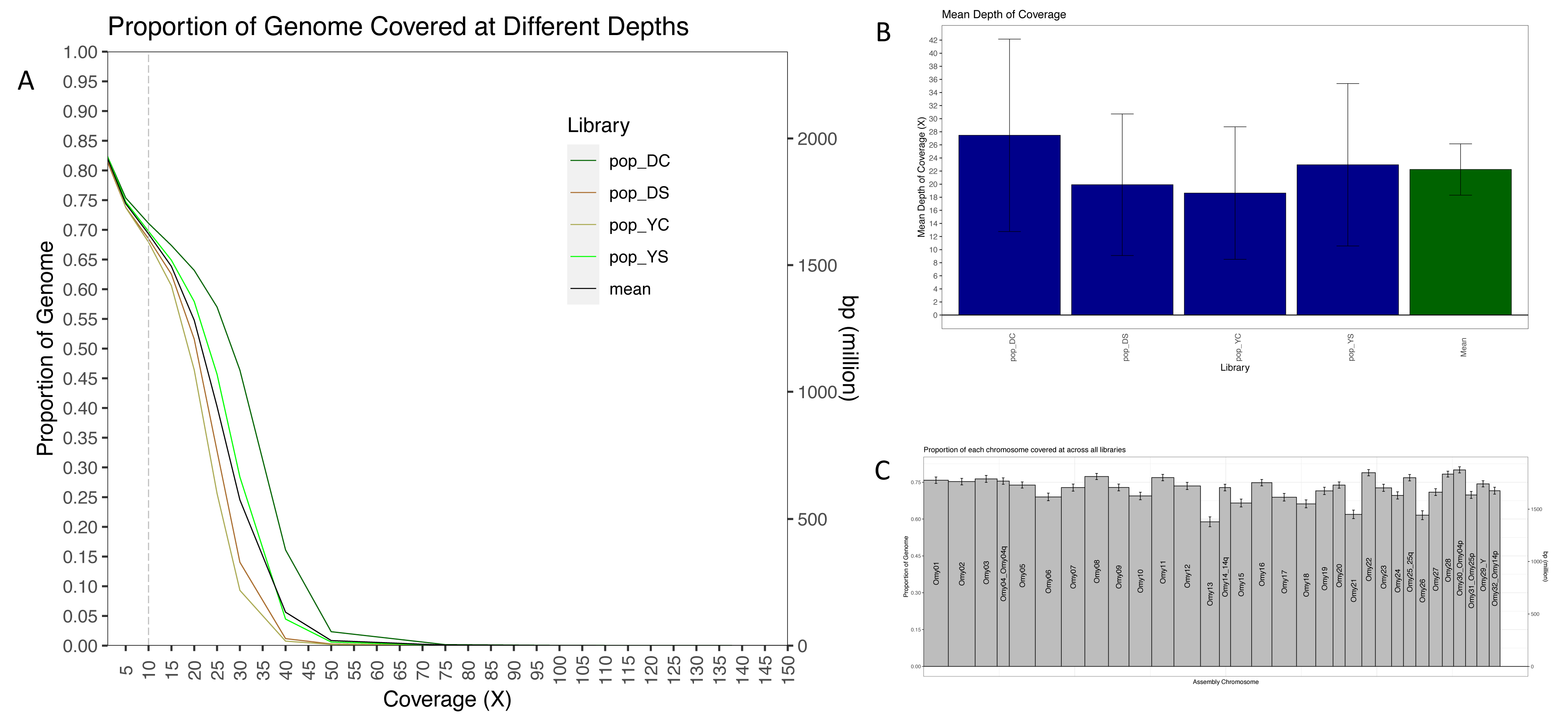

Supplement: Supplementary file 1 — Figure S1. [file EVA-17-e13622-s007.tif]

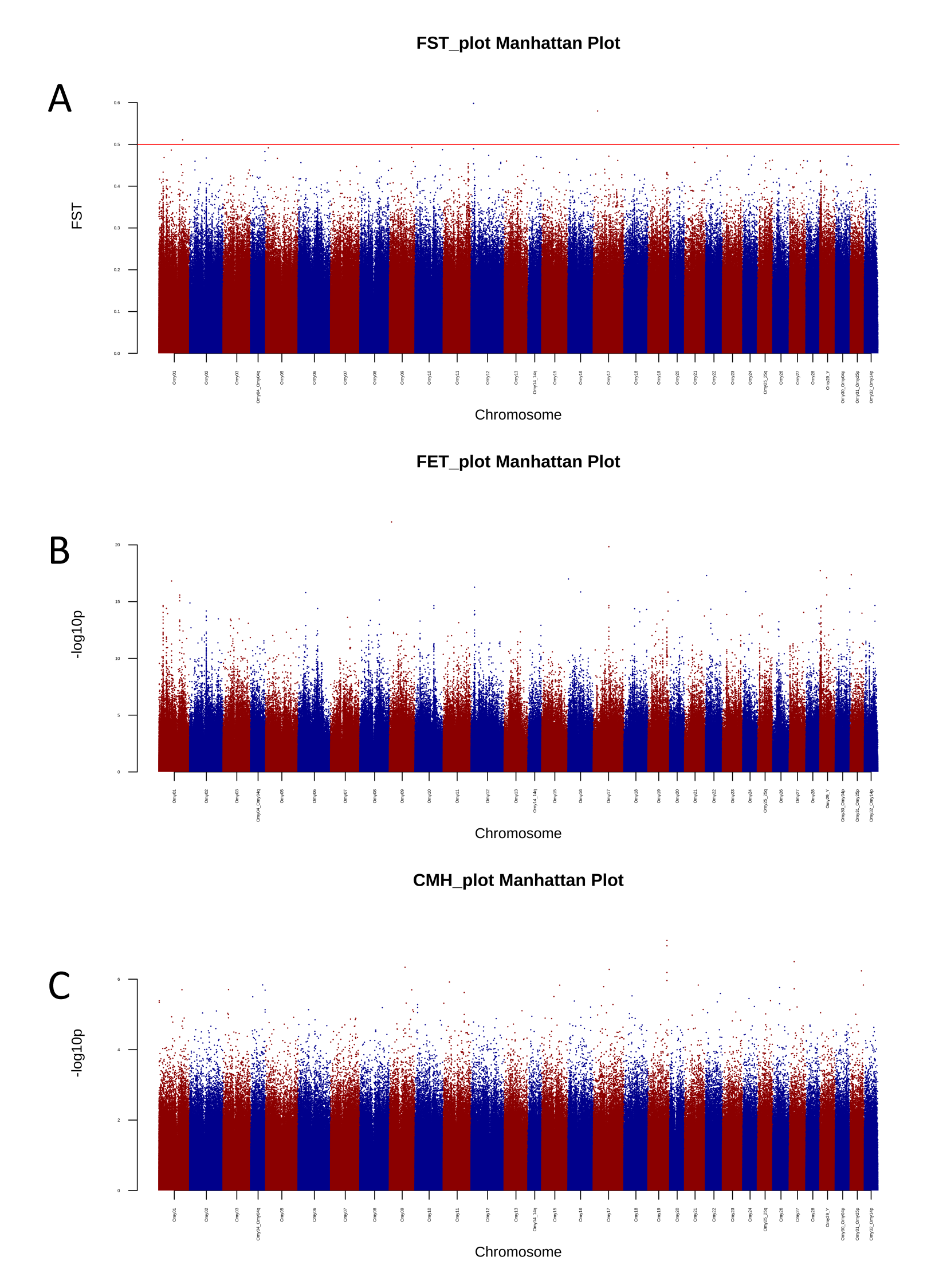

Supplement: Supplementary file 2 — Figure S2. [file EVA-17-e13622-s009.tif]

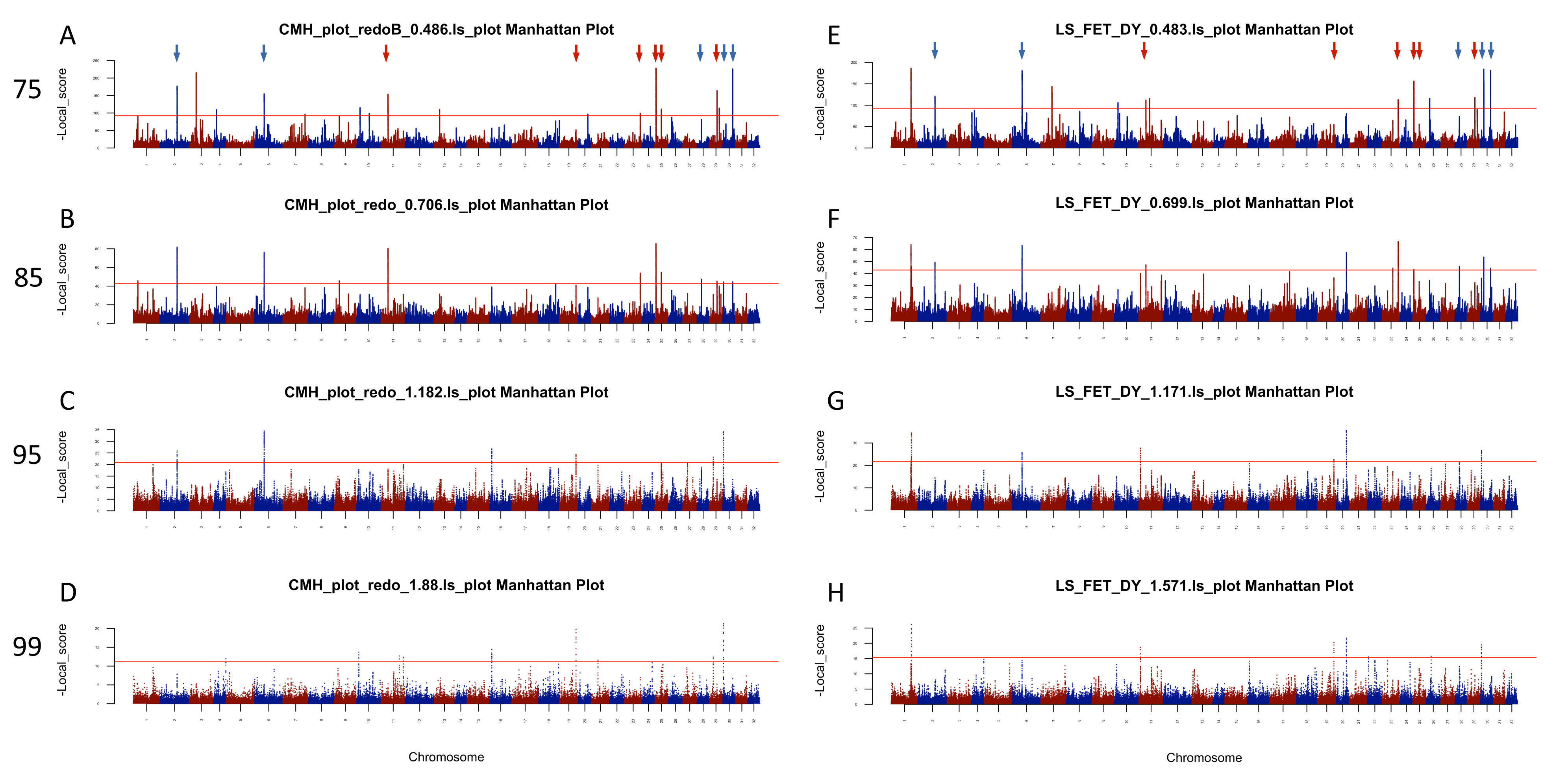

Supplement: Supplementary file 3 — Figure S3. [file EVA-17-e13622-s001.tif]

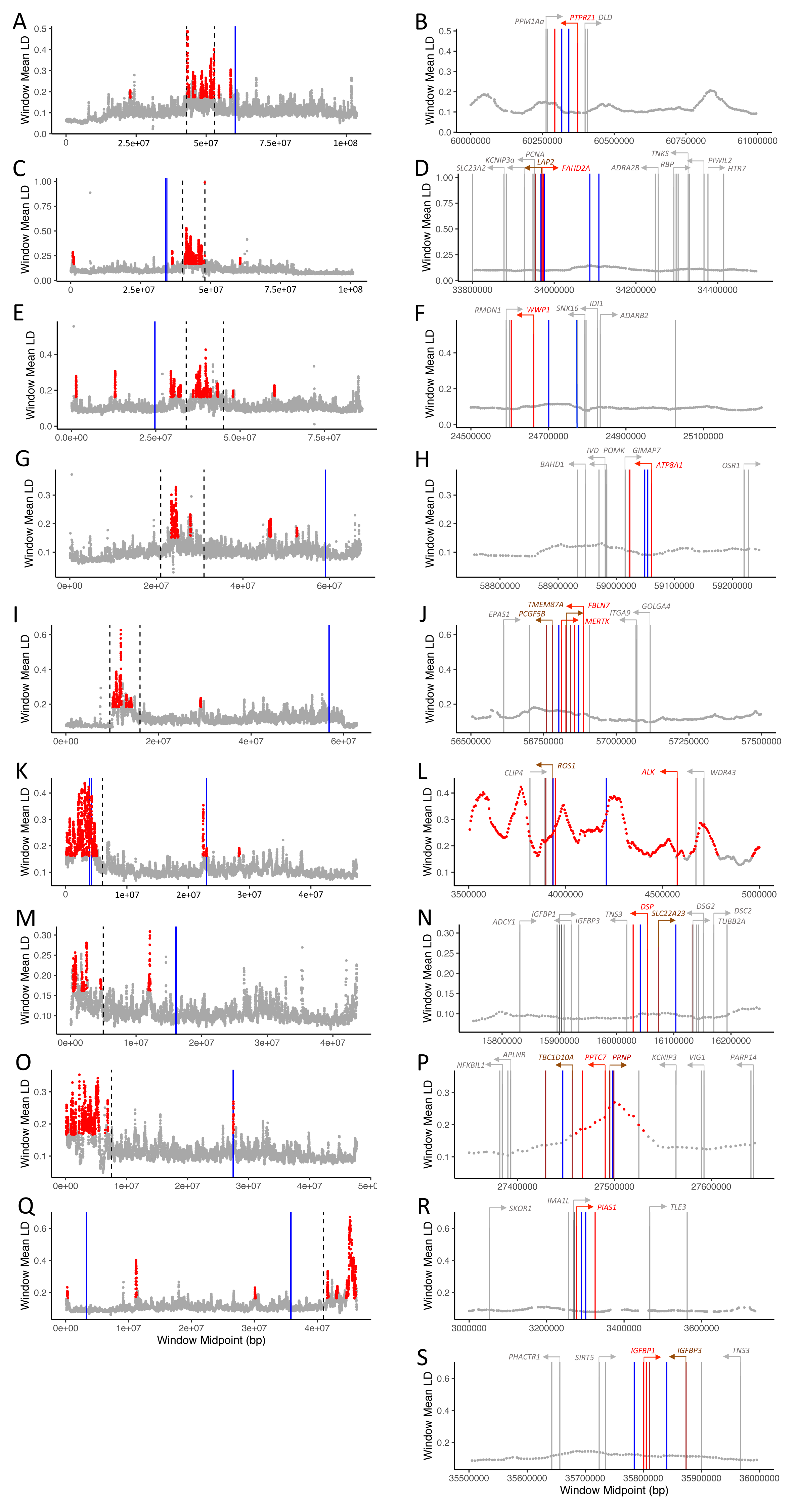

Supplement: Supplementary file 4 — Figure S4. [file EVA-17-e13622-s003.tif]

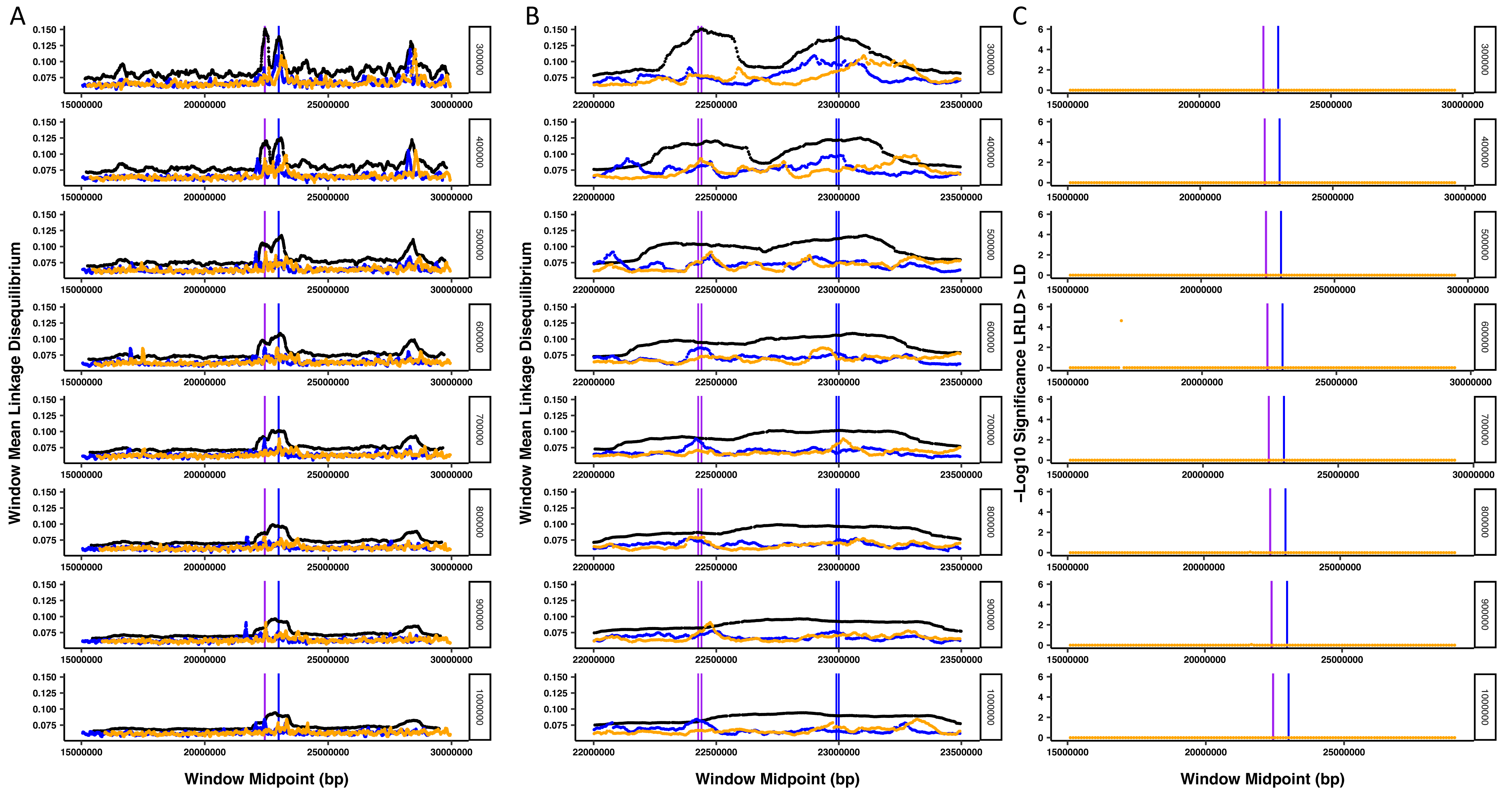

Supplement: Supplementary file 5 — Figure S5. [file EVA-17-e13622-s014.tif]

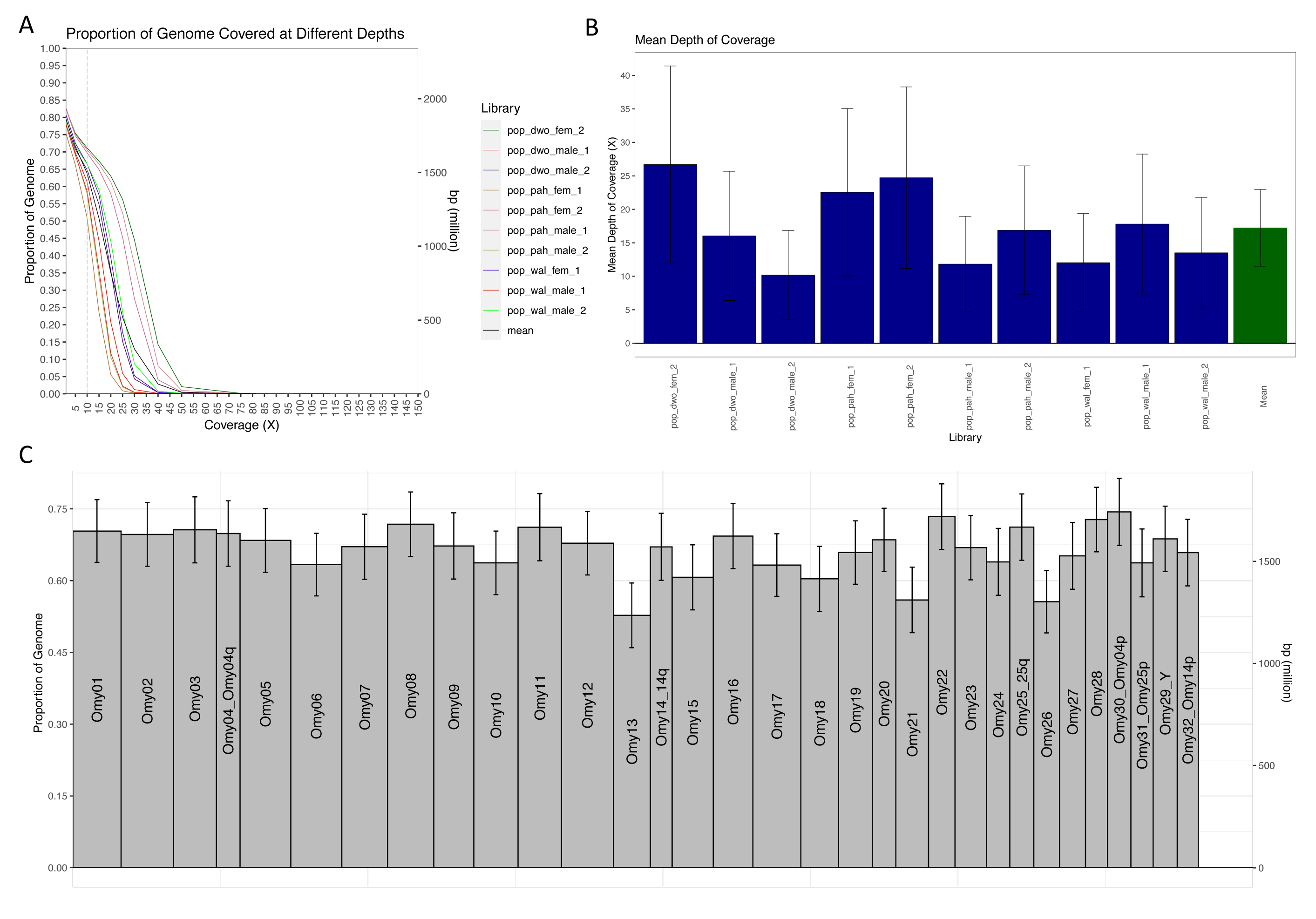

Supplement: Supplementary file 6 — Figure S6. [file EVA-17-e13622-s013.tif]

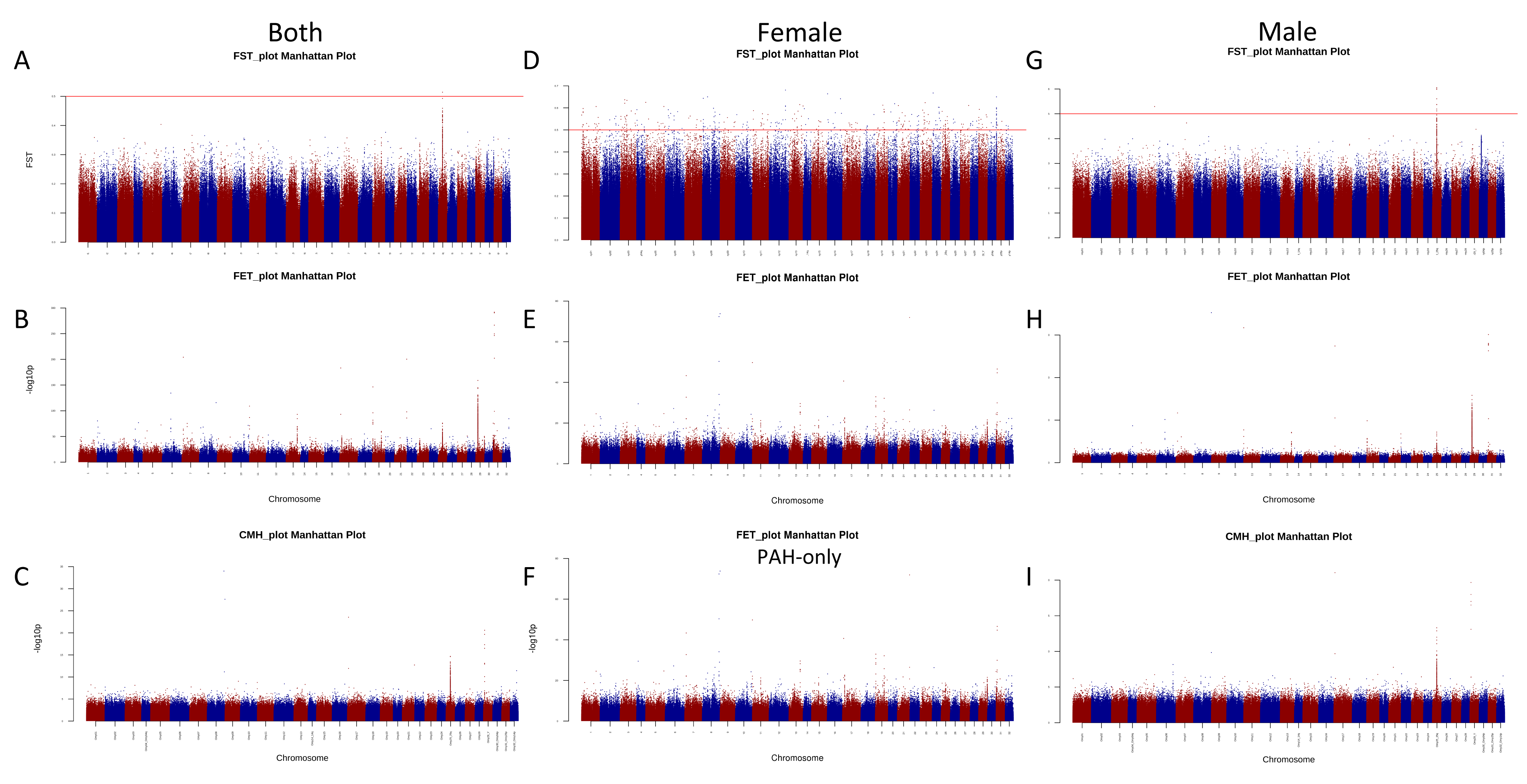

Supplement: Supplementary file 7 — Figure S7. [file EVA-17-e13622-s005.tif]

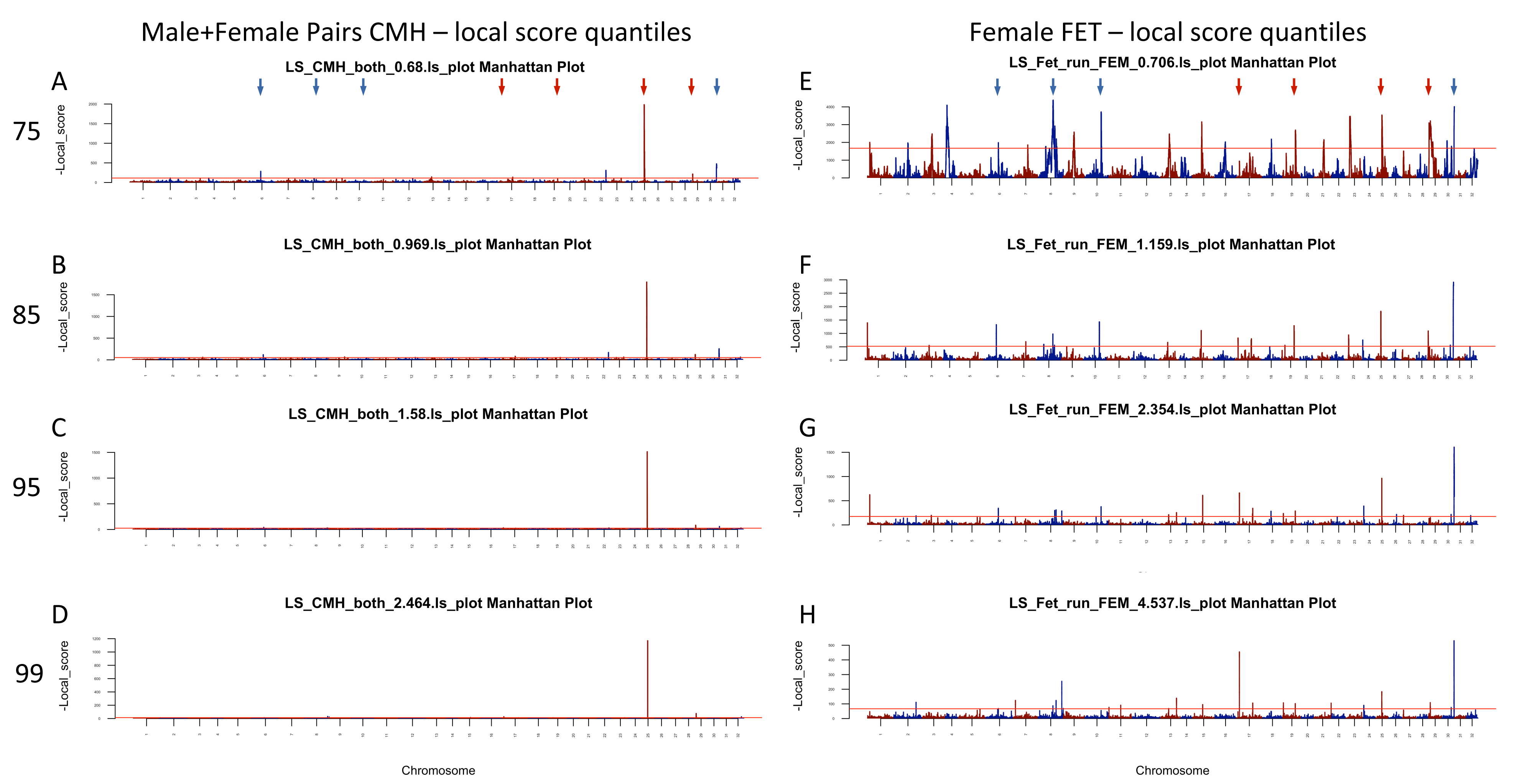

Supplement: Supplementary file 8 — Figure S8. [file EVA-17-e13622-s002.tif]

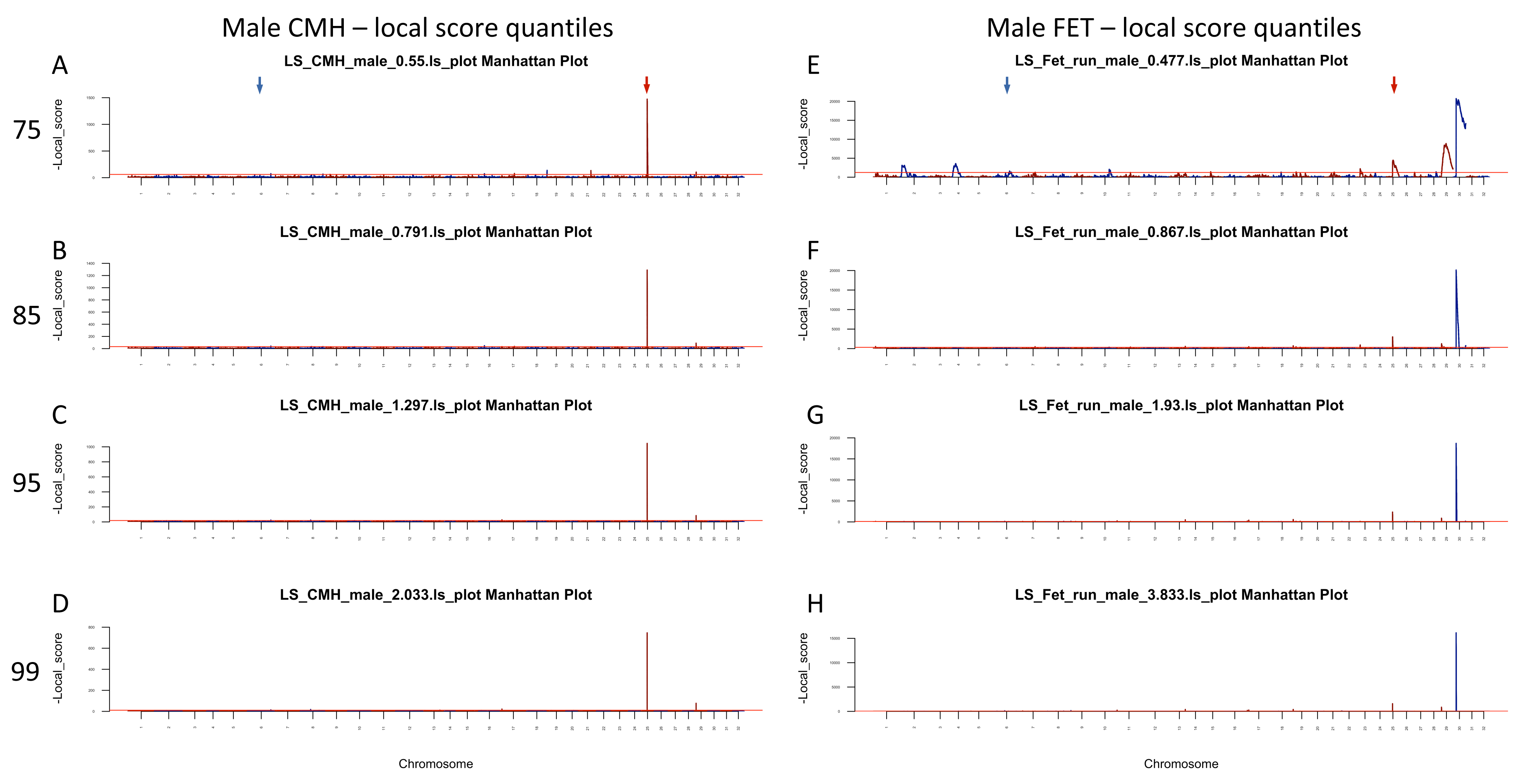

Supplement: Supplementary file 9 — Figure S9. [file EVA-17-e13622-s006.tif]
