# Supplementary material for: A genomic region associated with iteroparous spawning phenology is linked with age‐at‐maturity in female steelhead trout
Source: Evol Appl. 2023 Dec 11;17(2):e13622. doi: 10.1111/eva.13622 (PMC10853659; doi:10.1111/eva.13622)
Supplement: Supplementary file 14 — Data S1. [file EVA-17-e13622-s004.docx]

Supplemental File 1. Computer code to execute ANGSD, ngsLD, and summarize linkage estimates in sliding windows.

Supplemental File 2. Outlier peak boundaries identified by local score analyses of FET or CMH tests of association with iteroparous phenology and gene annotations and allele frequencies of highest scoring SNPs in select regions.

Supplemental File 3. Outlier peak boundaries identified by local score analyses of FET or CMH tests of association with age at maturity and gene annotations in select regions.
